# Can AI teach me employability? A multi-national study in three countries

**DOI:** 10.3389/frai.2024.1461158

**Published:** 2024-11-18

**Authors:** Dev Aditya, Krizia Silvestri, Pauldy CJ Otermans

**Affiliations:** ^1^Otermans Institute, London, United Kingdom; ^2^Division of Psychology, Uxbridge Brunel University of London, London, United Kingdom

**Keywords:** conversational AI, AI teacher, MOOC platform, AI in higher education, LLM for teaching

## Abstract

This paper examines the impact of using an Artificial Intelligence (AI) teacher for current Higher Education (HE) students from three countries. The study utilized an AI avatar powered by a fine-tuned Large Language Model (LLM), OIMISA, which is trained solely for teaching and learning applications. The AI teacher provided a 9-lesson course on employability and transferable skills. In total 207 students across the three institutions enrolled in the programme. The results demonstrate a noteworthy completion rate of over 47%, along with high levels of engagement across all student cohorts and high satisfaction rates from the students. These show the potential for AI-based virtual teachers across countries for students of HE compared to the use of MOOC platforms.

## 1 Introduction

In recent years, Artificial Intelligence (AI) has made significant strides in education (Jian, 2023). The integration of AI into educational systems promises to revolutionize the way students learn and interact with educational content. Traditional classroom settings, while effective, often face challenges such as limited personalized attention, varying learning paces among students, and resource constraints (Gamalel-Din, 2017). Asynchronous learning environments have been traditionally utilized to support learning outside the classrooms and to support some of these challenges (Jorgensen, 2012). These systems allow learning outside the classroom with low reliance on time and space of learning. In asynchronized learning environments students watch pre-recorded content, read materials and engage in quizzes and activities set by teachers. These platforms, although conceptually promising, have traditionally suffered from low engagement and completion rates (Jordan, 2015; Duncan et al., 2022). The concept of AI teachers offers a potential solution to these challenges by providing personalized, scalable, and consistent educational experiences specifically in Higher Education (HE) (Pratama et al., 2023). These AI teachers fall under the subgroup of pedagogical agents and have been previously used in different pedagogical use cases like conversational tutors and coaches (Winkler and Söllner, 2018). The integration of AI in the teaching process presents several challenges and limitations. One of the main concerns is the lack of emotional intelligence in AI systems, which limits their ability to provide empathetic feedback and adapt to the emotional states of students (Luckin and Holmes, 2016). Additionally, AI tools often face issues related to data privacy and security, particularly when handling sensitive student information (Shum and Luckin, 2019). The effectiveness of AI-driven teaching also heavily depends on the quality and diversity of the training data, and biases in the data can lead to unequal learning outcomes (Holmes et al., 2019). Finally, teachers may lack the necessary skills and resources to integrate AI effectively into the classroom, creating a digital divide (Zawacki-Richter et al., 2019). This paper aims to address a few of these using its own AI system.

This paper examines a multi-national study that assesses the impact of employing an AI teacher to enhance learning beyond the conventional classroom in an asynchronized setting in three different countries from three continents. For the purpose of this paper, the AI teacher system is primarily compared against a benchmark of Massive Open Online Courses or MOOC platforms for asynchronous learning. This is because MOOC platforms have been mentioned as the dominant asynchronous learning system in HE environments (Setiadi et al., 1987). They have also seen a significant increase in use by top global HE institutions (Samim, 2018). Notably, since the COVID-19 pandemic, MOOC platforms have cemented themselves as being a very widespread tool in HE (Samoilenko, 2020). Finally in AI-enabled teaching, the incorporation of AI chatbots and AI enabled teaching assistants (Calabrese et al., 2022) into MOOC platforms and also scaffolding AI agents (Tegos et al., 2021) into such platforms has recently shown promising results. This can be because of their ability to increase engagement since such AI systems can make unidirectional learning on asynchronous platforms two-way conversational. Furthermore, some research suggests that repeated and increased engagement can lead to higher completion rates (Sunar et al., 2016).

The primary aim of this study is to understand how AI teachers can influence student engagement, increase course completion rates and gain a view on wider student satisfaction. The AI teacher used in this study is powered by OIMISA, a specialized Large Language Model (LLM) designed exclusively for teaching and learning applications. This is supported by recent research. Firstly, it has been evidenced that personalization offered by AI-driven platforms led to between 2- and 2.5-times higher learning gains (St-Hilaire et al., 2022). Although learning gains are wide in scope, research has also pin-pointed that these systems see an increased course completion rate compared to traditional MOOCs platforms (Yu et al., 2017; St-Hilaire et al., 2022). Adaptive AI enabled teaching can also use structures like contextual bandits in their design. These contextual bandits are reinforcement systems that help with personalization of content (Belfer et al., 2022). When such structures are used, these have led to improved student engagement and significant increase in course completion (Belfer et al., 2022). Similarly, the ability to use AI-based robots and characters to increase interactions in asynchronous learning environments has been suggested for online courses of HE subjects like economics (Lin et al., 2018). This ability of AI is not very subject specific or narrow as such suggestions have also been used for wider learning areas (Garimella and Varma, 2023) and different subjects like design as in the famous MERLIN research (Neo et al., 2022). In this research, students were presented with a multimedia-based AI chatbot named MERLIN. Results on student’s attitudes toward using MERLIN showed that students were more motivated to learn more using MERLIN, improved their learning, and wanted more chatbots in other courses. Similarly, using an AI teacher to teach non-HE students single courses has been tried (Aditya et al., 2021). However, the utilization of a topic agnostic AI teacher for an entire course across different groups of HE students has been limited. Additionally, the use of an AI teacher for employability skills training of HE students, which are universally required skills for all HE students, has not been explored. These are the focus of this paper.

Additionally, research has explored students’ perceptions of AI teaching assistants and found that students perceived them useful because of the ease of communication with the AI assistants and it was a key factor in their increased adoption (Kim et al., 2020). Therefore, this study will test whether the AI teacher will lead to higher engagement among learners, higher completion rates and improved student satisfaction as compared to the benchmark of MOOC platforms.

## 2 Methods

### 2.1 Materials

**Figure 1:**
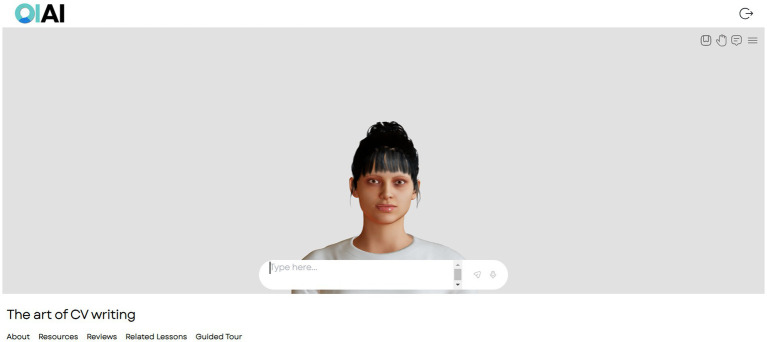
The AI teacher interface.

**Figure 2:**
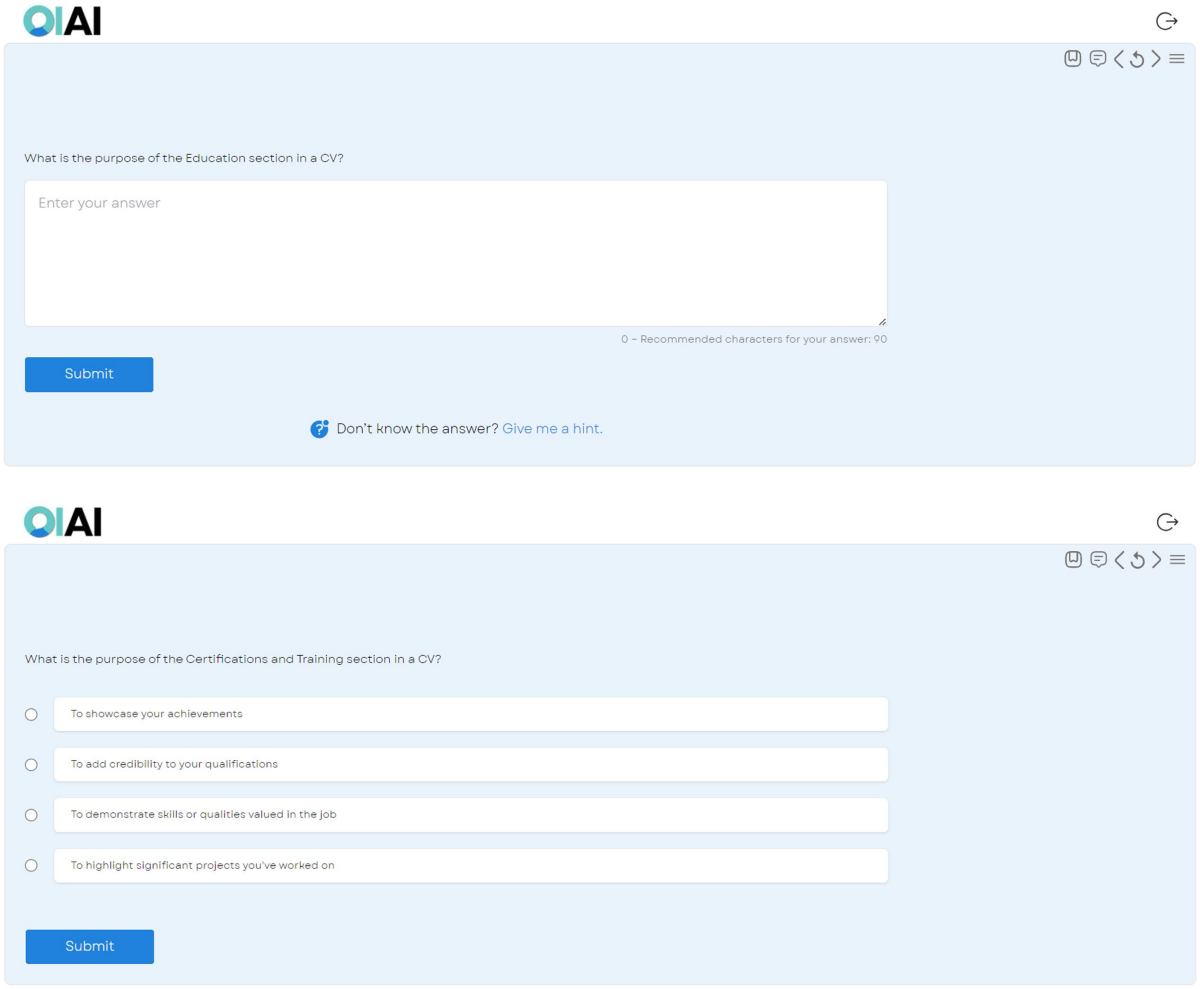
The AI teacher quizzing the student on the content. Top: an example of an open-ended question. Bottom: an example of a multiple-choice question.

The AI teacher is an AI-powered digital teacher that is available as a web-based application which students can log in to without the need of downloading a special application. This AI teacher can support students during their entire learning journey of the set course (Figure 1). It teaches them the specific skills of the course while also quizzing them on the content (Figure 2). The AI teacher can also listen to their voice and understand their questions, giving them tailored support and answers to their individual questions in real time. Where students do not want to speak to the AI teacher, they can also communicate with it by typing.

The AI teacher is available 24/7 during the programme. Learning with this AI teacher has many features, such as:

- Students can learn at their own pace, time, and place, without any pressure or judgment.
- Students can ask all their questions and get answers immediately.
- Students can have fun and engaging learning experiences, with interactive activities and quizzes.
- Students can get feedback and guidance from the AI teacher based on their performance on the quizzes.

**Figure 3:**
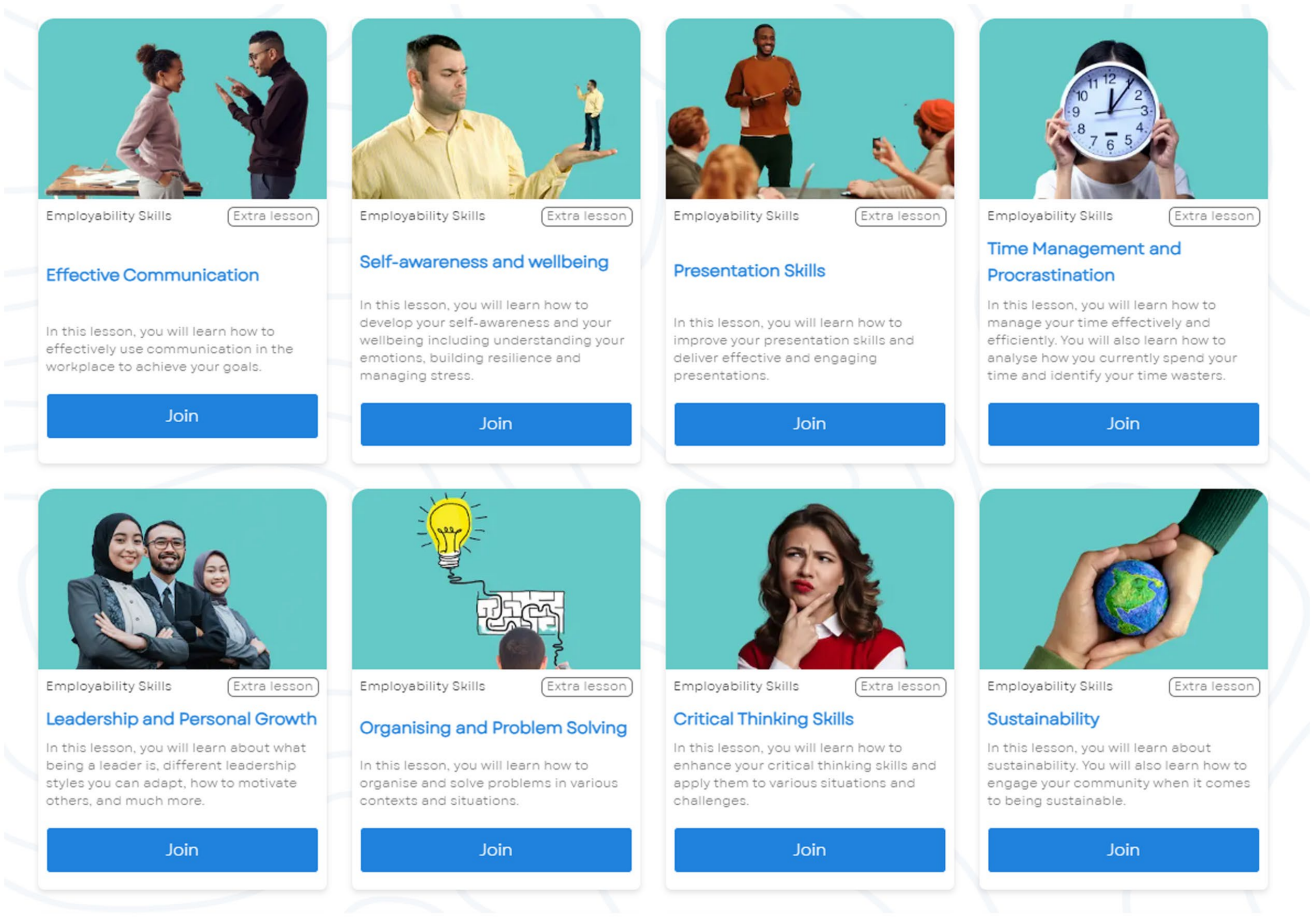
The interface showing how students accessed all nine lessons.

Students engaged with the AI teacher that was ingested with an employability and transferable skills course which covers the essential skills HE students need for their career. Employability skills were chosen as the content of the programme because of the rising acceptance of embedding employability within HE (Matherly and Tillman, 2015) with some academics also calling it the heart of HE (Bennett et al., 2015). Finally, a recent study found that while online learning on employability skills did not have a higher completion rate compared to average completion rates of online courses, it did have a slightly higher enrolment rate (Novella et al., 2024). In this research, students could access the lessons anytime and anywhere from any smart device (laptop/PC/tablet/phone). The course consisted of nine lessons, each with approximately 1 h of interactive learning and quizzes. The topics of the course were the following (Figure 3):

- Goal setting and motivation
- CV writing
- Effective communication skills
- Self-awareness and wellbeing (Including social media use)
- Presentation skills
- Time management and procrastination
- Leadership and personal growth
- Organizing and problem solving
- Critical thinking

### 2.2 Technical set-up

The technical design of the AI teacher is an important component of this study. The AI teacher was developed using OIMISA, a specialized fine-tuned LLM tailored for educational purposes. This was done to have control over the interface design and the content generated by the AI teacher using an existing course; all of the teaching content of the nine lessons and the quizzes that the students experienced were generated by OIMISA. This generation of content and quizzes by the AI is different from plugging in a chatbot onto an existing MOOC platform where the content was created by humans. With OIMISA-7B, the human is always in the loop. The human uploads the document that they want the learners to learn. Then the AI extracts the information from the document and generates the teaching script activities. The human can check the script that OIMISA-7B generates and make any edits where needed. Also, the human chooses the type of activities to be used during the learning, in this case, multiple choice question and open questions, which are then generated by OIMISA-7B. Again, the human can check and edit the questions and answers generated by OIMISA-7B before releasing the lesson to the learners.

The AI teacher interface was designed to mimic a human teacher’s interactions as closely as possible. Unlike a copilot style interface, which assists users mainly in completing tasks (Prather et al., 2023), the AI teacher interface was created to provide a comprehensive learning experience. Key features included:

1. Avatar representation: The AI teacher was represented by an avatar, making interactions more engaging and relatable for students.
2. Interactive topic: Each topic of the course included interactive elements (i.e., quizzes).
3. Personalized answers: The AI teacher provided personalized answers to questions asked by students during the learning process.

**Figure 4:**
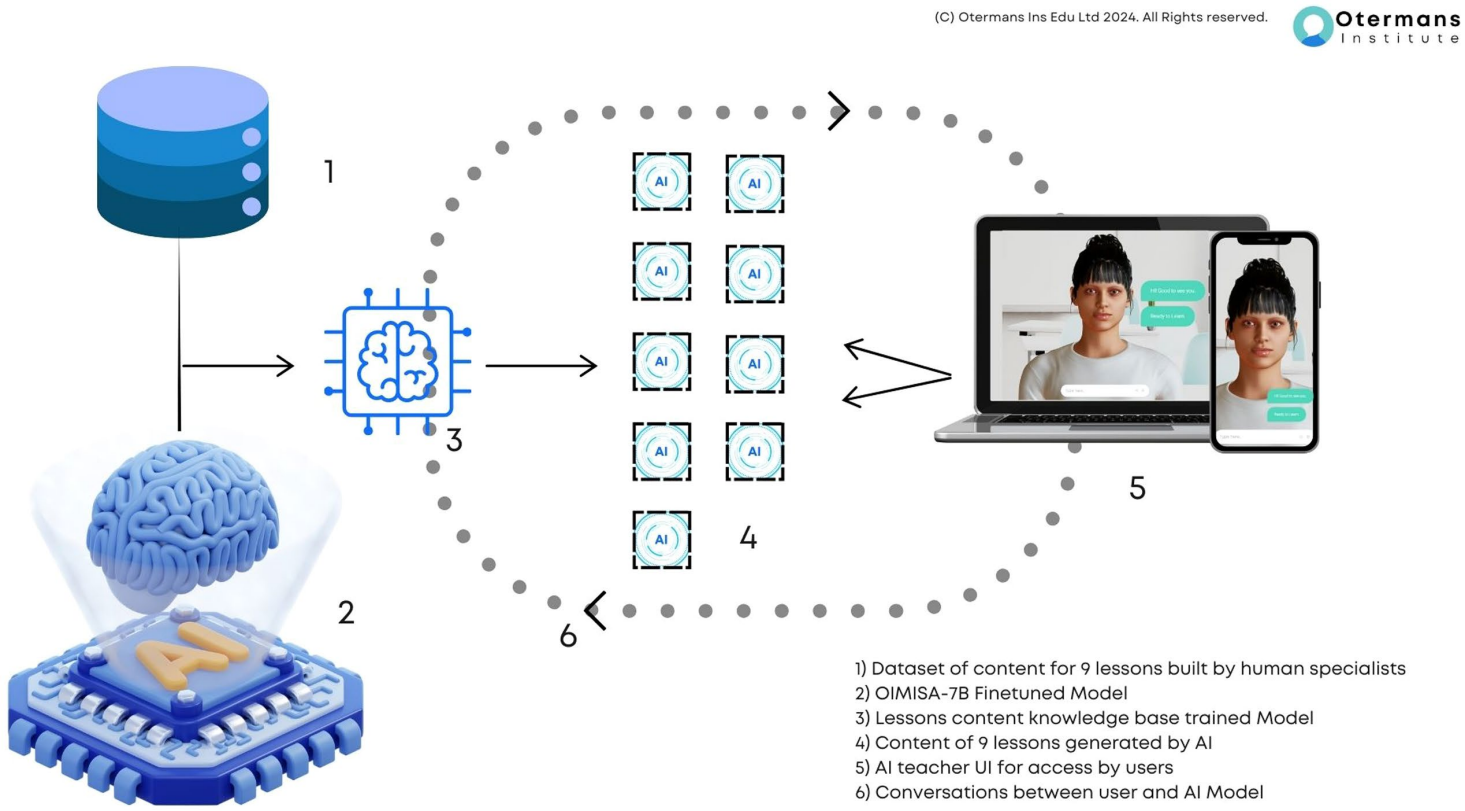
The OIMISA model.

The fine-tuned LLM (OIMISA) was specifically designed for teaching and learning applications. An important reason for using this model was its focus on teaching delivery and it being trained on the specific contents of an existing employability and transferable skills course (Otermans and Aditya, 2022). Key attributes of OIMISA include (Figure 4):

- *Domain-specific training:* The model was trained on a corpus of content from an employability and transferable skills course which comprised of the 9 topics being taught. The course was previously used to teach students from more than 8 countries by human trainers (Otermans and Aditya, 2022). This ensured high accuracy and relevance of the teaching content which the AI system generated.
- *Natural language understanding*: OIMISA was capable of understanding and responding to student queries in natural language, making interactions seamless. This was achieved by developing the model using the Transformer based system which enables LLMs to function (Luo et al., 2023). The model was trained on a corpus of data encompassing teaching delivery content, ensuring high relevance and accuracy on answer quality when students asked it questions. Additionally, the fundamental linguistic ability of OIMISA was developed by adding the MISTRAL 7B open source LLM model to the training process as it is a high efficacy open source LLM with good language capabilities (Jiang et al., 2023).
- *Data anonymization:* All private data were anonymized. The data themselves were taken after institutional approval.
- *A/B testing with bias checks:* Controlled experiments where responses from the AI were tested against different population groups were done prior to rolling out the AI teacher to the larger student group to identify potential biases.
- *Bias of AI responses:* The authors regularly audit AI responses through diverse human review panels to identify any unintended biases in the AI responses. This was done through our custom-built analytics dashboard that recorded every single interaction (anonymized) and samples of AI responses were checked weekly.

### 2.3 Participants

For this study, students from three universities from three separate continents took part in a 9-lesson programme. A total of 112 students enrolled from Arab American University (Institution 1), which is an institution in Palestine, Asia. From IBN Haldun University (Institute 2), a university in Turkey, Europe, 70 students enrolled. Finally, 25 students enrolled from Universidad de Las Americas (Institute 3), a university in Chile, South America. Apart from their university, no demographic data was collected from the participants. Participants were randomly selected by the host institutions. Students had 24/7 access to the AI teacher for a period of 5 weeks between November 2023 and January 2024. The three student groups represented varied demographic groups from three different continents. These groups also had separate native language and cultures. This helped give a balanced sampling (although each group was not of the exact same size). Similarities in the groups that we required was understanding of using smart devices like smart phones, tables and laptops, as well as being accustomed to learning in English. Therefore, multiple language options were not provided. This meant there was no need to translate content into the local language and we did not need to undergo cross-cultural adaptation process.

### 2.4 Data analyses

The data were analyzed using IBM SPSS Statistics version 28 (IBM Corp, 2021).

### 2.5 Procedure

Students would receive an email with all the detailed instructions and the link to access the course. After receiving the link, they needed to complete their registration. Upon verification of the registration, they could start joining the lessons. Students could complete the lessons in their own time and pace, and had 5 weeks to complete all nine lessons. As part of their induction, a webinar was held where students were guided through the use of the AI teacher. In addition to receiving the recording of the webinar, they also received a short pdf with guidance including screenshots. They received no other support.

## 3 Results

As the primary aim of this study was to understand whether AI teachers can influence student engagement and increase course completion rates, these results are presented first.

### 3.1 Student engagement

**Figure 5:**
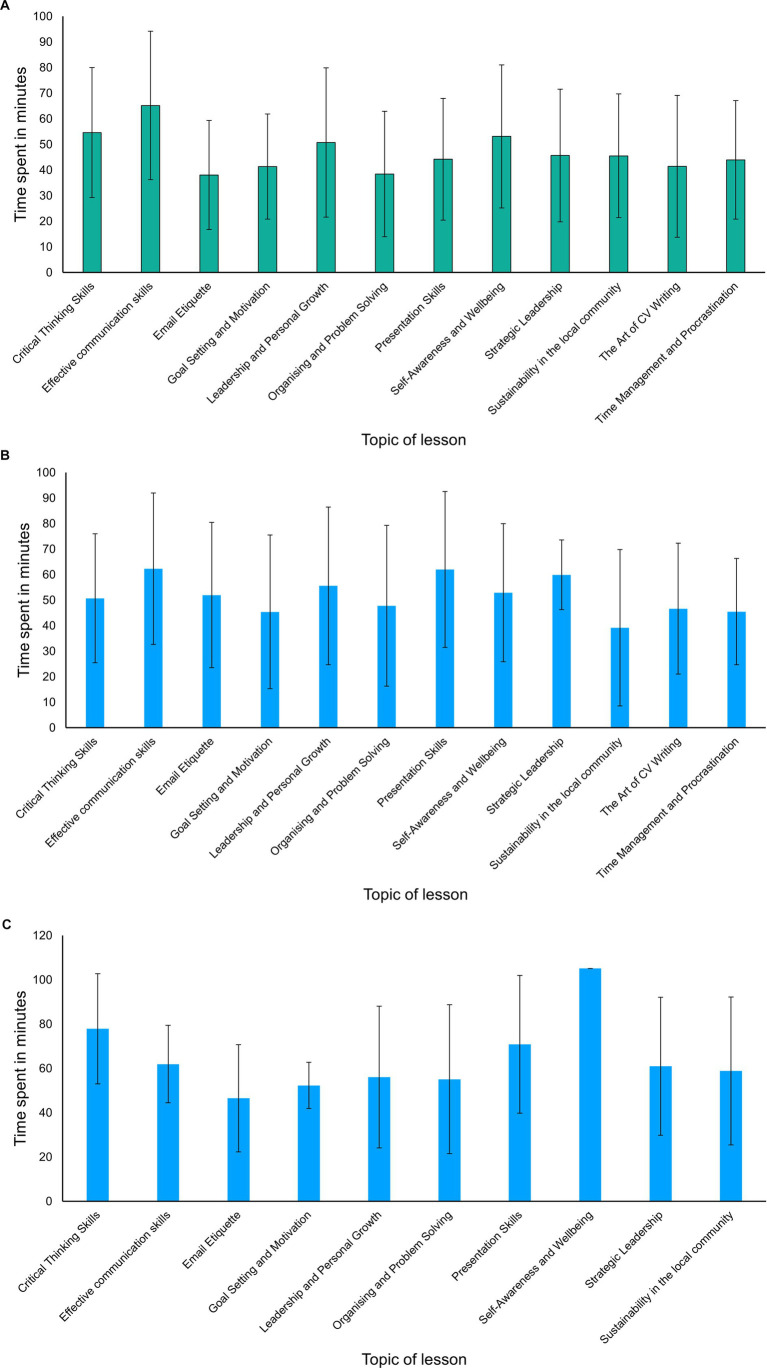
(a) Average time spent in minutes per lesson by students of Institute 1. (b) Average time spent in minutes per lesson by students of Institute 2. (c) Average time spent in minutes per lesson by students of Institute 3.

As this was an exploratory study and the first-time students engaged with an AI teacher, student engagement with the AI teacher was measured by the time they spent per lesson and the number of questions asked to the AI teacher, and any extra lessons joined voluntarily beyond the 9-lesson programme that they were enrolled in. The data were checked for outliers. Datapoints indicating more than 120 min spent per lesson were discarded in this analysis. This is because the content cannot be more than 1 h (as set by the AI) and we double this for slow learners and for those who engage more through the questions and answers with the AI. In terms of time spent per lesson, Figures 5a–c show the time spent per lesson in minutes per institute. Each lesson was designed to take approximately 50–60 min to complete. For Institute 1, students spent more than 60 min for each lesson apart from the lesson on Time management and procrastination. This shows significant engagement with the content of the programme.

A similar pattern was observed for Institute 2. Here, students spent more than 60 min for each lesson apart from the lesson on Goal setting and motivation. This again shows significant engagement with the content of the programme.

A slightly different pattern was observed for Institute 3. Here, students spent on average more than 50 min for each lesson with the lowest being 47 min for the lesson on Goal setting and motivation. This again shows significant engagement with the content of the programme.

A second measure of student engagement was the interactions with the AI teacher which was measured by the number of questions asked by the student to the AI teacher. Students could interrupt the AI teacher at any time by clicking the “raise hand” button and ask their questions for clarifying, doubt clearing or deep diving of the content. This is not possible with static content that institutions may ask them to go through as part of independent study where the institution can just measure whether someone clicked on that content. For Institute 1, students asked 449 unique questions in total. A similar pattern was observed for Institute 2 where students asked 456 unique questions in total. The number was slightly lower for Institute 3, where students asked 153 unique questions in total but it is important to note that there were significantly fewer students enrolled from Institute 3. Some example interactions between the students and the AI teacher are provided below:

Student: “What language should I use when writing a CV profile?”; AI: “When writing a profile for a CV, it is important to use language that is professional and concise. Avoid using slang or jargon, and focus on using words that clearly and accurately communicate your skills and experience. Additionally, make sure to use appropriate grammar and punctuation” - Interaction between student from Arab American University and AI on the topic of CV writing.

Student: “I want to learn how can I start to thinking like critcal?”; AI: “Great! I’m happy to help you start thinking critically. Let us start by understanding what critical thinking is and why it is important. Can I explain that to you?” - Interaction between student from Ibn Haldun University and AI on the topic of Critical Thinking.

Student: “How can I use a productivity app to stay focused?”; AI: “That’s a great question, [Student name]! Productivity apps can be a great way to stay focused. You can use them to track your progress, set reminders, and keep track of tasks. You can also use them to set goals and stay motivated.” – Interaction between student from UDLA and AI on the topic of Time Management and Procrastination.

**Figure 6:**
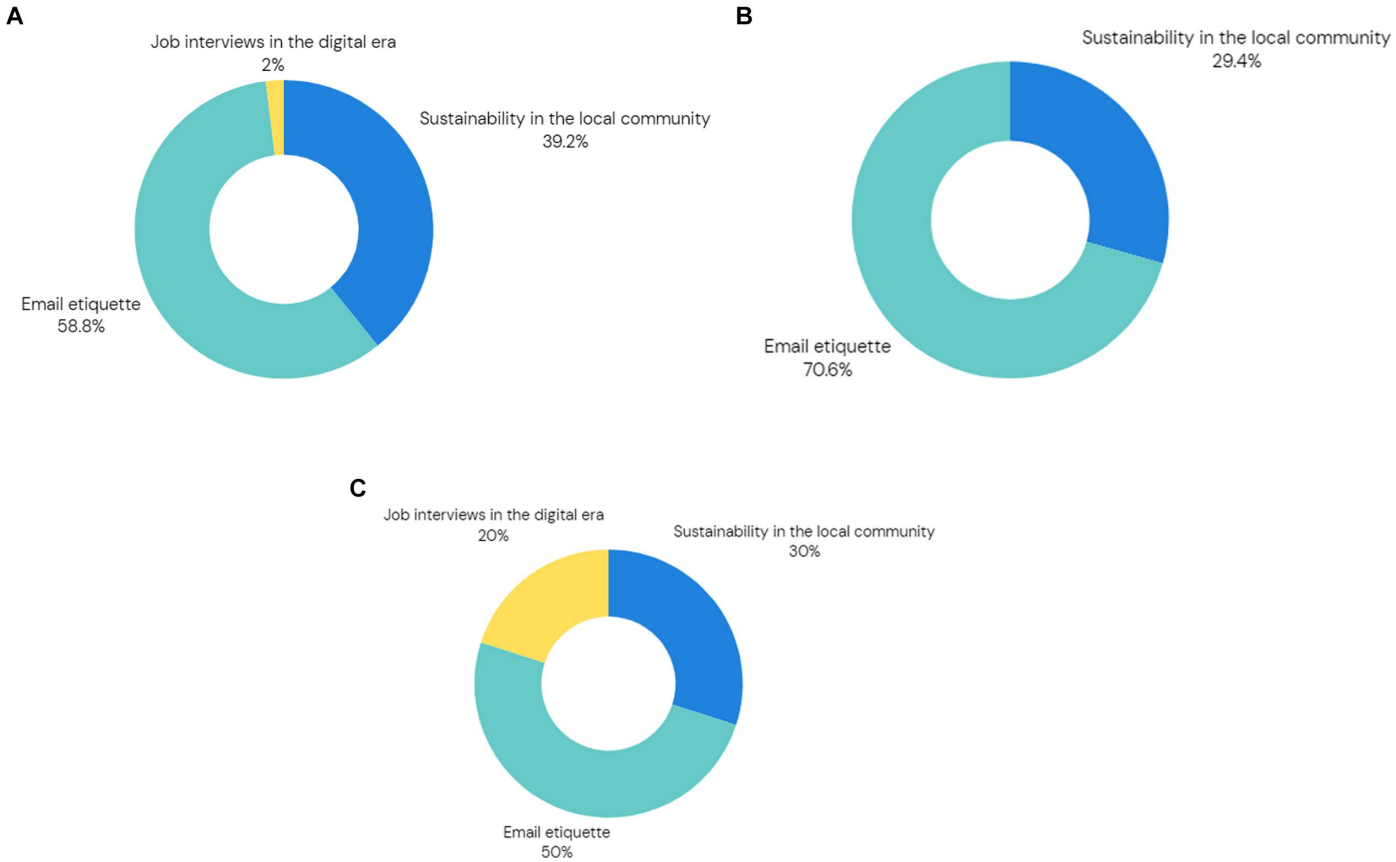
(a) Distribution of extra lessons joined by students from Institute 1. (b) Distribution of extra lessons joined by students from Institute 2. (c) Distribution of extra lessons joined by students from Institute 3.

The final measure of student engagement was the number of extra lessons students joined voluntarily above and beyond the 9-lesson programme. There was no requirement or incentive for them to join extra topics. There were three extra topics: Job interviews in the digital era, Sustainability in the local community, and Email etiquette. For Institute 1, 62 (55%) students joined at least one extra lesson. Figure 6a shows the distribution of extra lessons joined highlighting the topic of Email etiquette being the most popular. This remarkable participation indicates the students actively sought additional knowledge and benefited from the pursuit of non-compulsory subjects.

For Institute 2, only 17 (24%) students joined extra lessons. Notably, none of them joined the topic of job interviews in the digital era (Figure 6b) and again Email etiquette was joined by the majority. Nonetheless, 24% is a large number of students who took advantage of joining an extra lesson and gaining extra knowledge and developing more skills.

For Institute 3, 10 (40%) students joined extra lessons, again a very large number of students. Figure 6c shows again that the topic of Email etiquette was the most popular one.

### 3.2 Course completion rates and satisfaction rates

The second area of interest for this study was the completion rate of the 9-lesson programme with the AI teacher. The completion rates were as follows: 64% (Institute 1), 47% (Institute 2), and 56% (Institute 3). Please note that this only includes those students who completed all nine lessons each with 100% completion rate. This shows high engagement with the 9-lesson programme.

**Figure 7:**
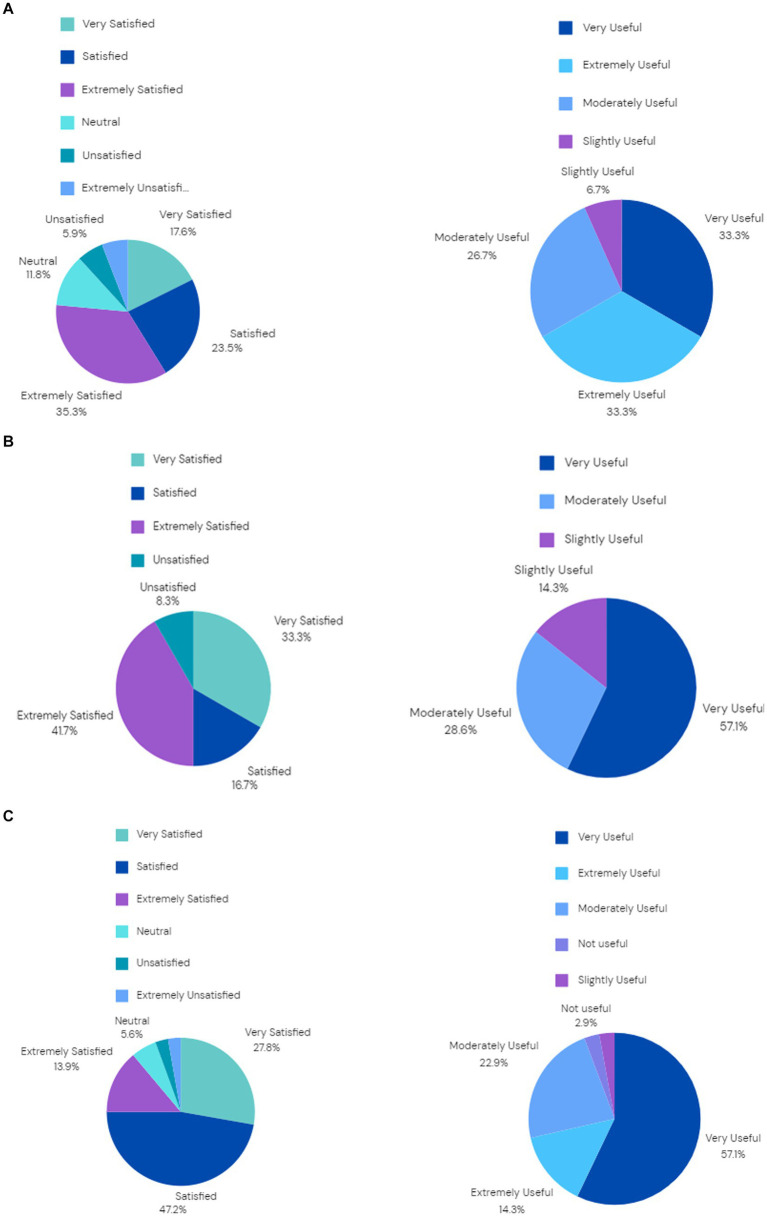
(a) Students’ overall satisfaction with the AI teacher (left) and usefulness of the AI teacher (right) for Institute 1. (b) Students’ overall satisfaction with the AI teacher (left) and usefulness of the AI teacher (right) for Institute 2.7. (c) Students’ overall satisfaction with the AI teacher (left) and usefulness of the AI teacher (right) for Institute 3.

A secondary aim of the study was to gain a view on wider student satisfaction of learning with an AI teacher. At the end of each lesson, students were asked to rate their overall satisfaction with the AI teacher using a 7-point Likert-type scale ranging from (1) Extremely unsatisfied to (7) Extremely satisfied. Similarly, they were asked to rate the usefulness of the lesson with the AI teacher using a 5-point Likert-type scale ranging from (1) Not useful to (5) Extremely useful. Figures 7a–c shows the overall student satisfaction and usefulness ratings for each of the three institutes. Figure 7a shows that for Institute 1 76.4% of students were satisfied (total of “satisfied,” “very satisfied” and “extremely satisfied”) with the lessons provided by the AI teacher. In addition, 66.7% (total of “very useful” and “extremely useful”) of students found the sessions with the AI teacher useful. Please note that no student rated the usefulness as “Not useful.”

Figure 7b shows that for Institute 2 91.7% of students were satisfied (total of “satisfied,” “very satisfied” and “extremely satisfied”) with the lessons provided by the AI teacher. It should be noted that no students rated their satisfaction as “very unsatisfied” and “extremely unsatisfied.” In addition, 57.1% (total of “very useful”) of students found the sessions with the AI teacher useful. Please note that no student rated the usefulness as “Not useful” but also no student rated the usefulness as “extremely useful.”

Figure 7c shows that for Institute 3 88.9% of students were satisfied (total of “satisfied,” “very satisfied” and “extremely satisfied”) with the lessons provided by the AI teacher. In addition, 71.4% (total of “very useful” and “extremely useful”) of students found the sessions with the AI teacher useful.

Students were also asked to provide any comments they had on the paper. Below are some quotes from participants on their learning experience with the AI teacher:

“It’s all good and I like this idea,” “No suggestions,” “it’s too much fast when cothe talking” – Students from Arab American University.

“Great teacher,” “It was a different experience but a nice one,” “The slides at left side can be shown accordingly to related part of the lesson. They are very informative and I want to see them” – Students from Ibn Haldun University.

“The AI speaks a little too slow.” – Students from UDLA.

## 4 Discussion

The primary purpose of the study was to explore whether AI teachers can influence student engagement and course completion rates. Results showed that for the three institutions, students spent more than 60 min for the majority of the 9 topics indicating significant engagement with the content of the programme. This is in line with previous research which has shown AI-powered interface designs to improve student engagement by up to 25.13% (Xu et al., 2023).

Students demonstrated a remarkable ability to communicate effectively with the AI teacher. This was evident by the large number of unique questions asked to the AI teacher for each institute again indicating significant engagement with the content of the programme. This facilitated an environment where they felt comfortable asking questions without fear of judgment, enabling them to seek clarification and explore concepts in greater depth. Even though per person interaction and engagement is traditionally low in MOOC platforms, these increased when there were larger cohorts in MOOC forums (Baek and Shore, 2016). This suggests that when students can clearly communicate (through the forum), their engagement with the course is higher. While the interactions using an AI-enabled platform and a MOOC platform cannot be compared directly, the ability to communicate with an AI teacher leads to high engagement as students can speak openly and without limitation to the AI. Furthermore, recent studies have shown that specialized LLMs can enhance learning engagement and increase student participation (Izquierdo-Domenech et al., 2024; Tanwar et al., 2024). This is in line with the selection and use of OIMISA, a specialized teaching and learning LLM, to power the AI teacher of this study.

Notably, the minimum completion rate of 47% across the three institutions stands out as exceptionally high compared to the global average of 7–10% for free online digital learning courses (Duncan et al., 2022). This can be slightly higher at 12.6% (Jordan, 2015). This shows a significant positive ability of the AI teacher to improve completion rates. Furthermore, research has shown there is a direct, negative correlation between course length and completion rates (Lee and Chung, 2019). Despite having a course length of nine lessons where students took, for the majority of the lessons, over an hour to complete each lesson, the completion rate did not seem to be affected by this. Future research could investigate the completion rate of bite-sized lessons delivered by the AI teacher. Additionally, it is worth noting that a significant number of students of each of the three institutions in three continents voluntarily decided to take and complete additional lessons. This self-enrolment of students demonstrates that they enthusiastically pursued supplementary knowledge and gained valuable insights from additional topics, without any pushing activities from the programme coordinator at their institution.

Finally, the overall satisfaction with the lessons provided by the AI teacher across the three institutions was 76.4% and higher. Additionally, at least 57.1% of students found the lessons with the AI teacher useful. While these are very high, it should be noted that participants of MOOC platforms, generally rate their experiences positively (Gil-Jaurena et al., 2017).

The use of AI in teaching & learning brings notable advantages that can revolutionize the educational experience. One of the most significant benefits is the ability of AI to personalize learning for each student. As was seen in this study, each student was able to ask their own questions tailored to their learning needs. Also, AI-powered systems can assess students’ progress in real-time, identify learning gaps, and adjust the curriculum to meet their individual needs (Holmes et al., 2019). This was also evident in this study where the AI teacher tested the students through MCQs and open-ended questions and also provided feedback when the student did not answer correctly. This level of personalization allows for a more tailored approach to education, fostering a deeper understanding of subjects and enabling students to learn at their own pace. Furthermore, AI can automate routine administrative tasks, such as grading and attendance tracking, freeing up educators’ time to focus on more impactful activities like mentoring and student engagement (Luckin and Holmes, 2016).

However, implementing AI in teaching & learning does not come without challenges. An important challenge is the lack of emotional intelligence in AI systems. While AI can excel at delivering factual content and analyzing student performance, it struggles to replicate the empathy and emotional support that human teachers provide. Emotional intelligence is critical for fostering a supportive learning environment, addressing student anxieties, and motivating learners (Luckin and Holmes, 2016). Without this emotional connection, students may feel less engaged or supported, impacting their overall learning experience. In this study, the AI teacher encourages and motivates students but this can be further enhanced. Moreover, data privacy and security are significant concerns when AI systems are handling sensitive student information. The potential for data breaches or misuse of personal information poses ethical risks that need to be carefully managed. This was done in this study as the data is immediately encrypted and then anonymized for aggregated analyses.

### 4.1 Recommendations

This study has shown the potential benefits of using an AI teacher to support students’ learning. This can be particularly beneficial for learning outside the classroom. At the moment, educators do not know what students learn outside the classroom. They may collect some information, e.g., has the student clicked on the materials on the Virtual Learning Environment or Learning Management System; or the students’ knowledge was tested through an online quiz. However, if students have questions about learning materials they need to either wait till the next session, email the human teacher and wait for a respond; they do not get an immediate answer. Through the use of this AI teacher, educators can direct insights into the learning of students outside the classroom. They can see what areas students know, which ones they are struggling with, any barriers they were facing during their learning and what questions they asked. These data can support the educators to provide more personalized support during contact hours. By interacting with the AI teacher, students can receive personalized support outside of learning hours and have the ability to clarify doubts, ask questions or delve deeper at the time they want to learn.

### 4.2 Limitations and future studies

No study comes without limitations. In this study, no demographic data were collected from participants. Motivation to join the programme, age, gender, area of study could all be variables that can correlate with the interactions with the AI teacher and can be explored in further studies. A larger sample size would also be beneficial to perform inferential statistics. The study focused on students’ engagement with an AI teacher on topics related to employability and transferable skills development. Although the AI teacher is designed and development to be content agnostic, future studies could explore which domains are suitable for this form or learning and which may be less so. Another area for future studies could be a direct or controlled comparison with a traditional MOOC platform or other pedagogical methods to provide a reference/benchmark on the effectiveness of the AI teacher.

## 5 Conclusion

Completion rates in online learning platforms such as MOOC have been traditionally low. The rise of AI in Higher Education has the potential to change this. This study used an AI avatar that taught an entire nine lesson programme to students across three countries. Results showed high student engagement indicated by the time spent on the lessons, the number of questions asked, and the number of extra lessons joined. Additionally, the course completion rates across the three institutions were high. In sum, this study showed that through the use of an AI avatar, high online student engagement and course completion rates can be achieved.

## Data Availability

The datasets presented in this study can be found in online repositories. The names of the repository/repositories and accession number(s) can be found in the article/supplementary material.
